# Source Identification of Heavy Metals in Surface Paddy Soils Using Accumulated Elemental Ratios Coupled with MLR

**DOI:** 10.3390/ijerph18052295

**Published:** 2021-02-26

**Authors:** Jie Ma, Yali Chen, Liping Weng, Hao Peng, Zhongbin Liao, Yongtao Li

**Affiliations:** 1 Key Laboratory for Environmental Factors Control of Agro-Product Quality Safety, Ministry of Agriculture and Rural Affairs, Tianjin 300191, China; majie@caas.cn (J.M.); penghao333ph@163.com (H.P.); liaozhongbin93@163.com (Z.L.); 2 Agro-Environmental Protection Institute, Ministry of Agriculture and Rural Affairs, Tianjin 300191, China; yongtao@scau.edu.cn; 3 Department of Soil Quality, Wageningen University, P.O. Box 47, 6700 AA Wageningen, The Netherlands; 4 College of Natural Resources and Environment, South China Agricultural University, Guangzhou 510642, China

**Keywords:** heavy metals, soil profile, ratio, multiple linear regression, source identification

## Abstract

Source identification of heavy metals in agricultural soils using small sample sizes, simple experimental procedures, and convenient analysis is urgently required. This study employed a simple source identification model using a visual comparison via radar plots, cluster analysis, principal component analysis, and a multiple linear regression model to determine the source of heavy metal pollution in soil samples from the Chang-Zhu-Tan urban agglomeration area of China. The elemental compositions of major pollution sources (atmospheric deposition, organic fertilizer, irrigation water, and tailings) were compared with soil samples from 11 study locations and the model was used to determine the relative contribution of different pollution sources at each sample site. The results showed that the model successfully calculated the contribution of different pollution sources at each site based on the pollution characteristics and contaminant transport rules of the region. The proposed method overcomes the requirement for extensive data and complex experimental procedures. Furthermore, the model can determine the source of heavy metal contamination in single or small plots, which is important for the prevention and control of heavy metal soil pollution and remediation at the plot scale.

## 1. Introduction

Heavy metals are difficult to degrade. Therefore, heavy metal-contaminated soil is a serious environmental issue [1] that can threaten soil ecosystems, water reservoirs, and human health [2]. Sources of heavy metals in soils can be natural or anthropogenic. Naturally derived heavy metals are mainly controlled by geological parent materials [3], while anthropogenic sources may enter the soil through atmospheric deposition, activities related to mining, fertilizer application, or sewage irrigation. Atmospheric deposition and the application of organic fertilizer are the primary causes of heavy metal pollution in soils throughout China [4]; however, there are many other possible sources. For example, activities related to mining can cause heavy metal pollution in the area around the mine [5,6]. On a larger scale, the atmospheric deposition of heavy metals from activities related to mining may be a source of heavy metal accumulation in farmland [7]. Furthermore, irrigation is a critical source of Hg (38.63%) and Ni (22.69%) in Zhejiang province. The irrigation fluxes of most heavy metals in Zhejiang are higher than the national average level [8,9]. Consequently, it is important to identify the source of heavy metal pollution in specific areas.

Numerous methods for identifying these sources of pollution have been used with varying degrees of success. Inventory methods require counting of the number of inputs and outputs to establish a source inventory [7,8,9,10]. Similarly, geographic information systems (GIS) and the spatial analysis of variations in heavy metal concentrations can be used to determine their sources [11]. The receptor model avoids the need to collect information on each pollution source and does not require clarification of the pollutant transmission process. Therefore, the receptor model is the most commonly used technology in current studies [12]. Relatively simple receptor models, such as clustering analysis (CA), factor analysis (FA), and principal component analysis (PCA), can identify the common characteristics of different components via classification or dimension reduction; however, accurate source contributions are difficult to achieve [13,14,15]. Positive matrix factorization (PMF) introduces uncertainty into the process of evaluating the quality and reliability of data points and then combines the uncertainty with concentrations to apportion the sources [16]. A critical component of PMF is the optimal determination of the number of factors [17]. Advanced statistical algorithms, including conditional inference trees, finite mixture distribution models, and random forests, have a relatively high analytical accuracy [12]. However, all of the above-mentioned methods, including inventory, GIS, and receptor modeling approaches, require large amounts of data and a high computational workload.

The isotope ratio method quantitatively distinguishes the contribution of pollution sources by comparing isotope ratios between samples and pollution sources. On account of their stable geochemistry, small sample size requirement, and high identification accuracy, the isotopes of Pb, Cd, Cu, and Zn have been used to identify the sources of heavy metals in soils [18,19,20,21]. The Pb isotope method is the most mature and widely used among these. Based on a simple binary model, the approximate contributions of Pb from vehicle emissions and coal to atmospheric dust were 78.1% and 21.9%, respectively, suggesting that the former was the more important source of Pb in atmospheric dust [18]. According to the results of Pb isotope analysis, approximately 21% of As in the soils was impacted by smelters in a geogenic origin As-contaminated area in Korea [21]. However, the pretreatment and analysis of isotope samples are complicated and expensive. Therefore, there are many obstacles to the broad implementation of this approach.

Integrated methods have previously been applied to take advantage of different approaches. For example, Zhang, et al. [22] successfully applied the PMF-PCA-geo statistical analysis technique to identify the sources of heavy metals in Shifeng District, China. Another study integrated three technologies—GIS mapping, multivariate statistical analysis, and isotope ratio analysis—to assess heavy metal pollution and source apportionment in the peri-urban agricultural soils of Zhejiang Province, China [23]. A different study suggested that the combined applications of PMF, GIS, and PCA was accurate, pragmatic, and effective for the source apportionment of heavy metals in the cropland soils of Baiyin District, Gansu Province, China [13]. Although the integration of multiple methods increases the reliability of source identification results, the computational workload thereof increases markedly. Therefore, a simple method with a convenient application needs to be developed. The principal component analysis-multiple linear regression (PCA-MLR) model is a relatively simple method that has been applied to the source identification of organic pollutants and heavy metals in aquatic systems and soils in China [23,24,25,26]. In comparison to the spatial analysis and receptor model, MLR requires relatively few samples and a reduced workload.

This study investigates the use of a relatively simple model for the source apportionment of heavy metal pollution based on a small sample size, simple experimental procedure, and convenient analysis method. First, the tendency of heavy metals to accumulate in surface soils was determined, after which the ratios of these for different heavy metals in the Chang-Zhu-Tan urban agglomeration were calculated and compared to pollution source data for the region, inspired by the isotope ratio method. Then, CA and PCA were used to investigate the relationships between the characteristics of the ratios of different sites and pollution sources. Finally, MLR was employed to analyze the contributions of the pollution sources at various sites.

## 2. Materials and Methods

### 2.1. Sampling Area

**Figure 1:**
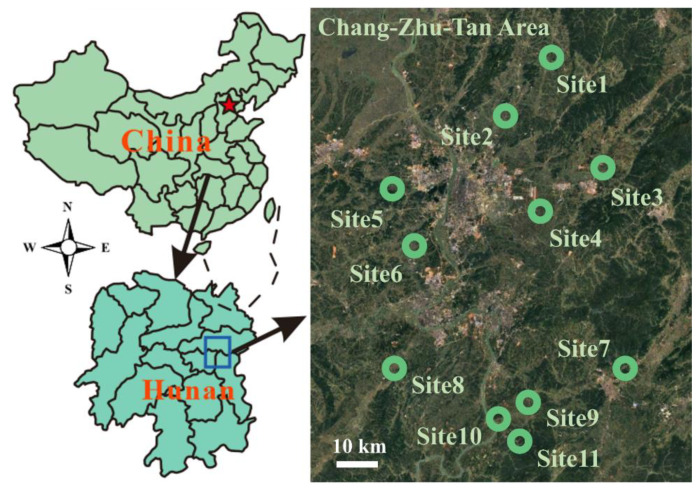
Locations of the sampling points investigated in the study.

The study site was located in the east-central region of Hunan province, China (left side of Figure 1). The location was comprised of the Changsha–Zhuzhou–Xiangtan (Chang-Zhu-Tan) urban agglomeration (left side of Figure 1) and the perimeter zones of Changsha, Zhuzhou, and Xiangtan, located along the middle reaches of the Yangtze River [27]. This urban agglomeration has a typical subtropical monsoon climate, with an average annual temperature of 18 ºC and annual precipitation between 1400 and 1700 mm [28]. The electricity, metallurgy, chemical, coal, building materials, textile, paper making, and food industries account for 33.75% of the total economy of the province. Crops are cultivated year-round, rice is the dominant cultivar, and crop rotation is barely practiced. The sources of heavy metals are diverse due to the complex industrial structure and large-scale agricultural production in the region.

### 2.2. Sampling and Data Collection

Eleven sampling sites of paddy soils were selected based on local farming habits and practices followed in the region in recent decades [29]. Of these, Site1 and Site2 were farmlands with normal applications of organic fertilizer, and Site3 was an area in which irrigation water was contaminated by livestock and poultry feces. Site4 was close to a road with heavy mining traffic. Site7, Site9, Site10, and Site11 were located in Liling City (under the jurisdiction of Zhuzhou City); Site7 was near an industrial area; and Site9, Site10, and Site11 were close to (approximately 10 km from) areas where lead-zinc mining occurred. Site8 was near an industrial area within Xiangtan City. Although potential pollution sources can be identified for these sites in the study area, the dominant source and its contribution could not be determined. The pollution sources at other sites (Site5 and Site6) were more ambiguous (Table 1).

The soil profiles in different paddy fields (Figure 1) were sampled at depths of 0–10, 10–20, 20–30, 30–40, 40–60, 60–80, and 80–100 cm using a soil core sampler. Stones and plant debris in the soil samples were removed by hand. All soil samples were air-dried and then ground to produce a fine powder (<0.074 mm) for further analysis. Soil samples were digested using HNO_3_–H_2_O_2_ [30] in a 50 mL glass digestion tube. The concentrations of elements (Cd, Cu, Pb, and Zn) were measured via inductively coupled plasma optical emission spectrometry (Optima 5300DV, Perkin-Elmer). Elemental recoveries in certified soil reference materials (GBW07307, Institute of Geophysical and Geochemical Exploration, Langfang, China) ranged from 92.5 ± 6.2% to 105.4 ± 5.3%. 

Data on Cd, Cu, Pb, and Zn in atmospheric deposition, organic fertilizer, tailings, and irrigation water were collected from 13 previous investigations which studied Chang-Zhu-Tan and its surrounding areas. Data on heavy metal concentrations in the atmospheric deposition (ng m^−3^), organic fertilizer (mg kg^−1^), tailings (mg kg^−1^), and irrigation water were collected from atmospheric particulates, livestock of pigs and chickens, mineral waste residue (-contaminated soil), and river water (μg L^−1^), respectively. In these studies, the samples were mostly digested with mixed acids. Although the determination method varied, each measurement was proceeded with quality control to make the data reliable. The average values and standard deviations are listed in Table 2. 

### 2.3. Data Analysis and Method of Source Identification

In this study, the concentration of a certain heavy metal that accumulated in surface soil (A_HM_) was calculated as the difference between the heavy metal concentration in the surface soil (S_HM_, average concentration of 0–10 cm) and in the bottom soil, which represented background values (B_HM_, average concentration of 60–80 and 80–100 cm). Therefore, A_HM_ is considered as the contribution from anthropogenic sources and the influence of soil parent materials is ignored in this study. The equation is given in Equation (1):

(1) $$A_{\mathrm{HM}}=S_{\mathrm{HM}}-B_{\mathrm{HM}}.$$

The ratios of two accumulated heavy metals (denoted as HM_a_ and HM_b_) in the surface soil (R_HMa/HMb_) were used to reflect the elemental characteristics of different sampling sites and evaluated according to Equation (2):

(2) $$R_{\mathrm{HMa}/\mathrm{HMb}}=\frac{A_{\mathrm{HMa}}}{A_{\mathrm{HMb}}}.$$

The ratio of two heavy metals (denoted as HM_a_ and HM_b_) from a particular pollution source (r_HMa/HMb_) were employed to determine the elemental characteristics of different pollution sources, as given in Equation (3):

(3) $$r_{\mathrm{HMa}/\mathrm{HMb}}=\frac{V_{\mathrm{HMa}}}{V_{\mathrm{HMb}}},$$

where V_HM_ is the heavy metal concentration in pollution sources (Table 2).

Radar plots of the 11 sampling sites and four pollution sources were then drawn based on the above R_HMa/HMb_ and r_HMa/HMb_.

Statistical analysis was conducted using the IBM SPSS for Windows, version 22.0. CA was applied to standardize data for hierarchical associations using Ward’s method for agglomeration and the squared Euclidean distance as a dissimilarity measure. Relationships among different pollution sources and sampling sites were investigated using PCA to reduce the correlation matrix to a minimum number of key factors. A scatter plot was prepared according to PC1 and PC2 to reflect the relationship among accessions in terms of pollution characteristics. The ratios of accumulated heavy metals were regarded as linear combinations of different pollution sources. Subsequently, MLR was performed on the significant factors to determine the apportionment of each source for specific pollution sites. Stepwise modeling was used to incorporate each independent factor (i.e., variable in the regression model) within the regression equation if it could significantly increase the correlation of the result, and a significant default level of 0.01 was used. After normalization, the MLR equation can be described by Equation (4):

(4) $$[R_{\mathrm{Zn}/\mathrm{Cd}},\;R_{\mathrm{Zn}/\mathrm{Cu}},\;R_{\mathrm{Zn}/\mathrm{Pb}},\;R_{\mathrm{Pb}/\mathrm{Cd}},\;R_{\mathrm{Pb}/\mathrm{Cu}},\;R_{\mathrm{Cu}/\mathrm{Cd}}{]}_{\mathrm{CZT}-i}^{T}\;=\;\sum \alpha_{\mathrm{PS}-j}[r_{\mathrm{Zn}/\mathrm{Cd}},\;r_{\mathrm{Zn}/\mathrm{Cu}},\;r_{\mathrm{Zn}/\mathrm{Pb}},\;\;r_{\mathrm{Pb}/\mathrm{Cd}},\;r_{\mathrm{Pb}/\mathrm{Cu}},\;r_{\mathrm{Cu}/\mathrm{Cd}}{]}_{\mathrm{PS}-j}^{T},$$

where [R_Zn/Cd_, R_Zn/Cu_, R_Zn/Pb_, R_Pb/Cd_, R_Pb/Cu_, R_Cu/Cd_]${\;}_{\mathrm{CZT}-i}^{T}$ is the column vector of the accumulated heavy metal ratios at the sampling sites (i = 11), and [r_Zn/Cd_, r_Zn/Cu_, r_Zn/Pb_, r_Pb/Cd_, r_Pb/Cu_, r_Cu/Cd_]${\;}_{\mathrm{PS}-j}^{T}$ is the column vector of the heavy metal ratios of the pollution sources (j ≤ 4). α_PS-j_ represents the unstandardized coefficients.

The standardized regression coefficient (β) can be derived from the coefficients above (α) using the following Equation (5):

(5) $$\beta_{\mathrm{PS}-j}=\alpha_{\mathrm{PS}-j}\frac{C_{\mathrm{PS}-j}}{C_{y}},$$

where C_PS-__j_ is the standard deviation of the independent variable, and C_y_ is the standard deviation of the dependent variable. β_PS-j_ represents the standardized coefficients and percentage contribution of different pollution sources. Although stepwise modeling can obtain different solutions, the one with the largest correlation coefficient is chosen.

## 3. Results and Discussion

### 3.1. Characteristics of Heavy Metals in Soil Profiles

**Figure 2:**
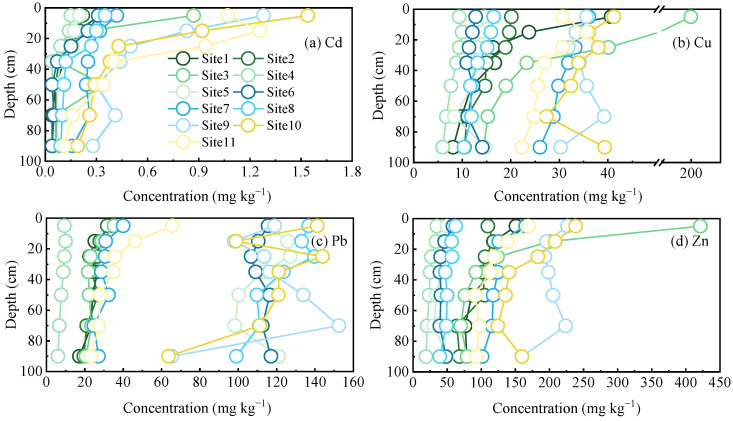
Concentrations of Cd (**a**), Cu (**b**), Pb (**c**), and Zn (**d**) in the soil profiles of different sampling sites.

The concentration of heavy metals decreased with depth in the soil profiles (Figure 2). Generally, the concentrations were highest in the surface soil (0–10 cm), and the average concentrations of Cd, Cu, Pb, and Zn were 0.61, 41.6, 76.8, and 153.4 mg kg^−1^, respectively. The concentrations of heavy metals were higher than those of the background soil values in the study area (Cd = 0.5 mg kg^−1^, Cu = 95.0 mg kg^−1^, Pb = 38.1 mg kg^−1^, and Zn = 60.3 mg kg^−1^), except for Cu [42], which showed accumulation of Cd, Pb, and Zn in the surface soil. Another study reported that from 2010 to 2013, the Cu concentration in the paddy soil in Changsha was very low (20.4 mg kg^−1^) [43], indicating the possibility of Cu accumulation in the studied area. The average concentrations of Cd, Cu, Pb, and Zn in the deep soils (60–80 and 80–100 cm) were 0.12, 17.5, 58.7, and 82.2 mg kg^−1^, respectively, and were significantly lower than those in the surface soils. Although an overall decreasing trend was visible, the rate of the decrement varied for different heavy metals across the sampling sites (Figure 2). There was a considerable decrease in the Cd concentration from the surface to deep soil (an average of 5.7 times); at five sites (Site1, Site3, Site6, Site10, and Site11), the magnitude of the decrement was more than six times greater (Figure 2a). Decreases in Cu concentrations with depth averaged 2.7 times greater, and only two sites (Site1 and Site3) had decreases greater than 3 times (Figure 2b). Decreases in Pb and Zn were lower than Cd and Cu and averaged 1.5 and 1.9 times greater, respectively (Figure 2c,d). Although Cd was transported downward in soil more easily than Cu and Pb [44,45], this action may have been slowed in the studied area due to the high content of clay minerals in the soil [46]. Similarly, a previous study has also shown that Zn tends to remain in surface soils [47]. Therefore, the higher concentrations of heavy metals in surface soils compared to deep soils indicate that heavy metals in the topsoil are derived from anthropogenic sources.

### 3.2. Classification of Potential Pollution Sources

**Figure 3:**
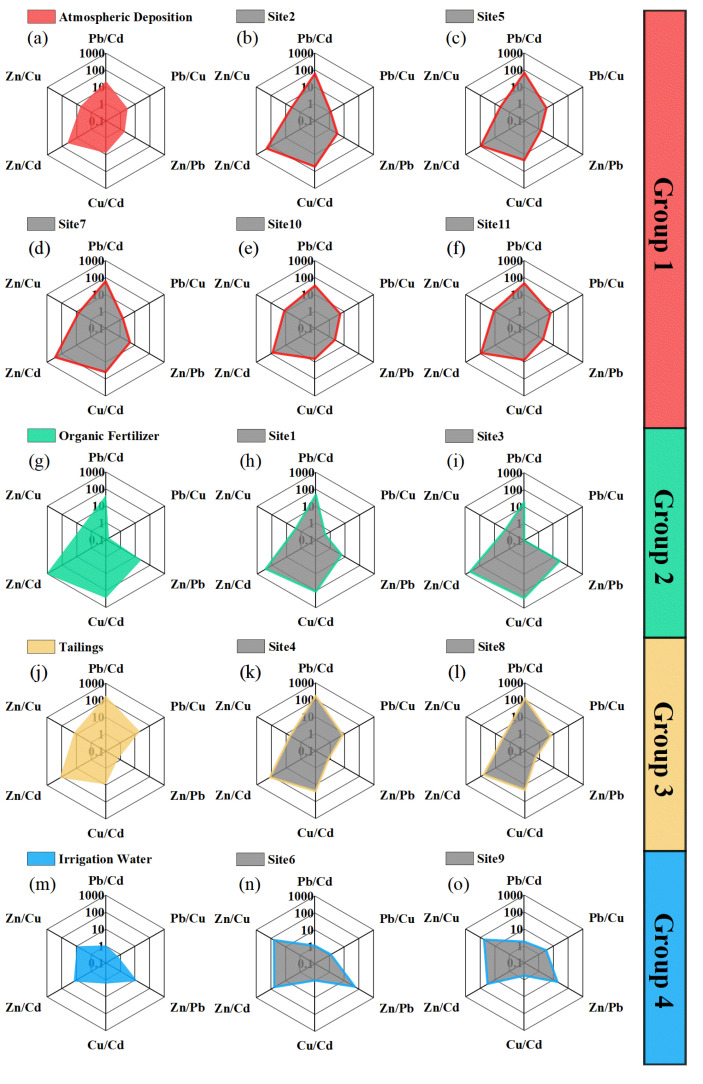
Radar plots of the ratios of heavy metals from different pollution sources (**a**, **g**, **j**, and **m**) and sampling sites (**b**, **c**, **d**, **e**, **f**, **h**, **i**, **k**, **l**, **n**, and **o**). Groups 1, 2, 3, and 4 reflect groups of sampling sites exhibiting similar radar plots as atmospheric deposition, organic fertilizer, tailings, and irrigation water, respectively.

Generally, Pb, Cd, Cu, and Zn are the dominant heavy metals derived from anthropogenic sources found in agricultural soils in both Hunan Province [7,48,49] and China overall [12,50]. Therefore, radar plots were used to visually compare the characteristics of these elements across sampling sites and pollution sources, as shown in Figure 3. The radar plots for the different pollution sources varied markedly. Atmospheric depositions contained concentrations of Pb (121.5 ng m^3^) and Zn (288.7 ng m^3^) that were over 10 times greater than that of Cd (5.28 ng m^3^) (Pb/Cd = 23.0 and Zn/Cd = 43.3) (Table 2 and Figure 3a). The relatively high concentration of Cu meant that the Cu/Cd (7.85) ratio was also high (Figure 3a). The radar plot of the atmospheric deposition showed a hexagon with small protrusions at the eight and twelve o’clock positions (Figure 3a). In organic fertilizer, the concentrations of Cu (166.4 mg kg^−1^) and Zn (671.9 mg kg^−1^) were 280.4 and 1132.8 times higher, respectively, than that of Cd. The Pb/Cu and Zn/Pb ratios (0.16 and 24.7, respectively) were relatively low and high, respectively, due to a low Pb concentration (27.2 mg kg^−1^) in the fertilizer (Figure 3g and Table 2). However, Pb concentrations that were high relative to Cd led to a much greater Pb/Cd value (45.9) (Figure 3g). The radar plot of organic fertilizer was concave at the two o’clock position and protruded at the four, six, eight, and twelve o’clock positions (Figure 3g). In tailings, the concentrations of Pb (8706.7 mg kg^−1^), Zn (6195.7 mg kg^−1^), and Cd (46.6 mg kg^−1^) were high due to the widespread distribution of Pb-Zn mining and smelting in Hunan province [51]. This resulted in high Pb/Cd, Pb/Cu, Zn/Cd, Zn/Cu, and Cu/Cd ratios of 187.0, 20.6, 133.7, 14.6, and 9.1, respectively (Figure 3j). The mining radar plot protruded at the two, eight, and twelve o’clock positions (Figure 3j). In the irrigation water, the Zn concentration (39.2 μg L^−1^) was 9.4 to 15.9 times greater than the concentrations of the other heavy metals (Table 2 and Figure 3m). The radar plot for irrigation water was hexagonal, with small protrusions at the four, eight, and ten o’clock positions (Figure 3m).

Therefore, the sampling sites exhibiting similar shapes in radar plots as the pollution sources were grouped. Group 1 (atmospheric deposition) consisted of Site2, Site5, Site7, Site10, and Site11; Group 2 (organic fertilizer) comprised Site1 and Site3; Group 3 (tailings) consisted of Site4 and Site8; and Group 4 (irrigation water) contained Site6 and Site9. Although the heavy metal concentrations of the pollution sources were uncertain (Table 2), a non-order of magnitude change in concentration would not alter the overall features presented in the radar plots.

### 3.3. Cluster and Principal Component Analyses

**Figure 4:**
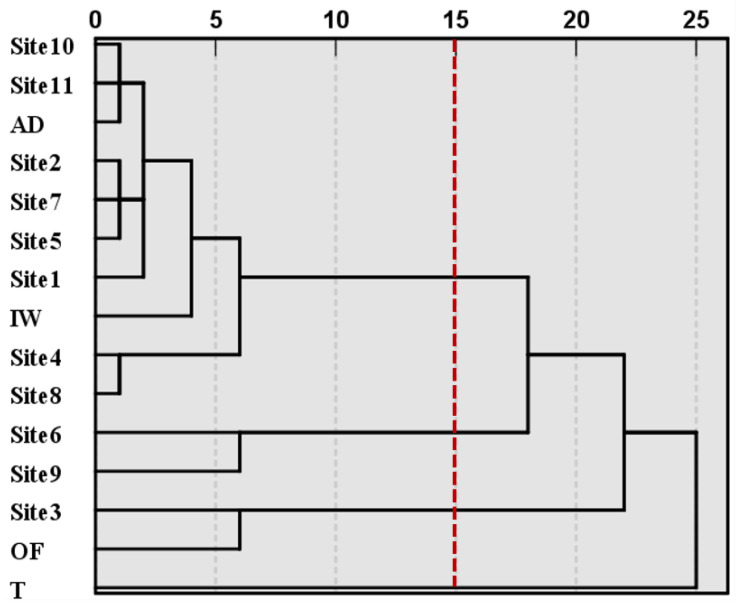
Dendrogram of the hierarchical clustering of the ratio of heavy metals accumulated at different sites. The pollution sources are atmospheric deposition (AD), irrigation water (IW), organic fertilizer (OF), and tailings (T).

The ratios of heavy metals accumulated at different sampling sites and the ratios of heavy metals for different pollution sources were processed using the CA to create four clusters of indices with similar properties, as shown in Figure 4. Cluster 1 included Site1, Site2, Site4, Site5, Site7, Site8, Site10, and Site11, and atmospheric deposition and irrigation water pollution sources. When compared to the previous groupings based on radar plots (Figure 3), Cluster 1 contained all sites of Group 1, in addition to Site1 from Group 2, Site4 and Site8 from Group 3, and irrigation water from Group 4. This indicated that other sampling sites and pollution sources (irrigation water) exhibited similar properties to Group 1. Meanwhile, the CA also confirmed that Group 1 had consistent characteristics. Cluster 2 included Site6 and Site9, suggesting that these two sites had a different elemental composition compared to irrigation water. Cluster 3 included organic fertilizer and Site3, indicating that that the pollution characteristics of Site3 were more affected by the organic fertilizer than Site1. Cluster 4 only included tailings, suggesting that the elemental composition of this source differed markedly from both the study sites and other pollution sources. However, this single cluster may have had a considerable influence on the CA results because hierarchical clustering is sensitive to outliers. The CA results differed markedly from those of the radar map classification; therefore, the relationship between the sampling sites and pollution sources requires further investigation.

**Figure 5:**
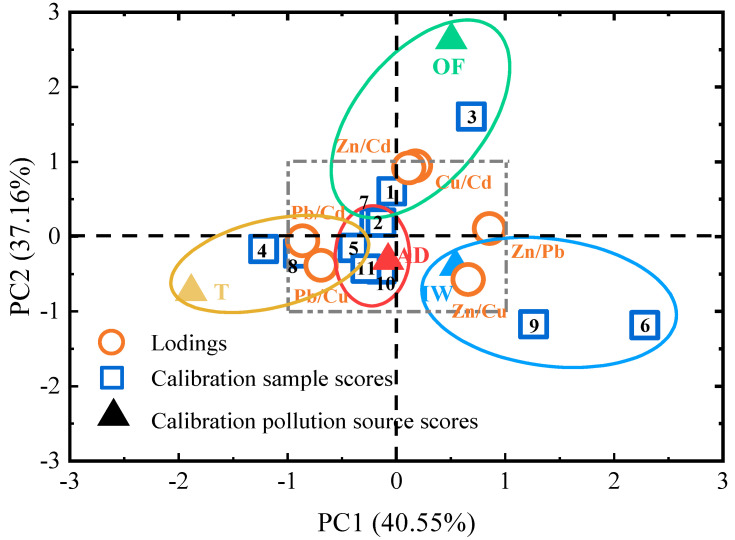
Correlations between the ratios of heavy metals and the first two principal components (PC1 and PC2) and a biplot of the ratios of heavy metals, sampling sites, and pollution sources (OF = organic fertilizer, T = tailings, IW = irrigation water, and AD = atmospheric deposition), according to the ratio of heavy metals accumulated in the latter.

In the PCA, two principal factors had a significant impact (77.71%) on the variance of the variables. Part of the variance in the first principal component (PC1, 40.55%) showed high positive factor loadings for Zn/Pb (0.856) and Zn/Cu (0.660), and high negative factor loadings for Pb/Cd (−0.861) and Pb/Cu (−0.692) (Figure 5). Copper and Zn are elements representative of agricultural activities; they make up approximately 69% and 51%, respectively, of total inputs from the application of livestock manure [9]. Lead, Cd, and Zn are characteristic elements of mining, particularly Pb-Zn mining, because Cd can enter into Zn-containing minerals via isomorphic substitution [52,53]. The different ratio relationships in PC1 further reflect the differences in elemental characteristics in agriculture and mining. The second principal component (PC2, 37.16%) showed high positive factor loadings for Cu/Cd (0.946) and Zn/Cd (0.918) and negative factor loadings for Zn/Cu (−0.572) and Pb/Cu (−0.386) (Figure 5). The fate of Cu and Cd in agricultural soils differs; the former is easily adsorbed and the latter is easily mobilized. Therefore, inverse relationships were observed in the ratio relationships when Cu and Cd were the denominators in PC2.

The distance between the data points and the pollution sources in Figure 5 reflects their correlation. To include all possible pollution sources in the linear regression performed on these data, the correlated groups were artificially enlarged and divided based on the quadrants they were located in and their distance from the origin. Site1, Site2, Site3, and Site7 in the first and second quadrant contained high concentrations of Cu and Zn and were classified as being related to organic fertilizer (green ellipse). Site1, Site2, Site4, Site5, Site7, Site8, Site10, and Site11 were classified in the second and third quadrants as pertaining to tailings (yellow ellipse). In turn, Site6 and Site9 were classified in the fourth quadrant as related to irrigation water (blue ellipse). Finally, Site1, Site2, Site5, Site7, Site10, and Site11 were classified close to the origin as pertaining to atmospheric deposition (red ellipse).

### 3.4. Identification of the Contribution of Pollution Sources

**Figure 6:**
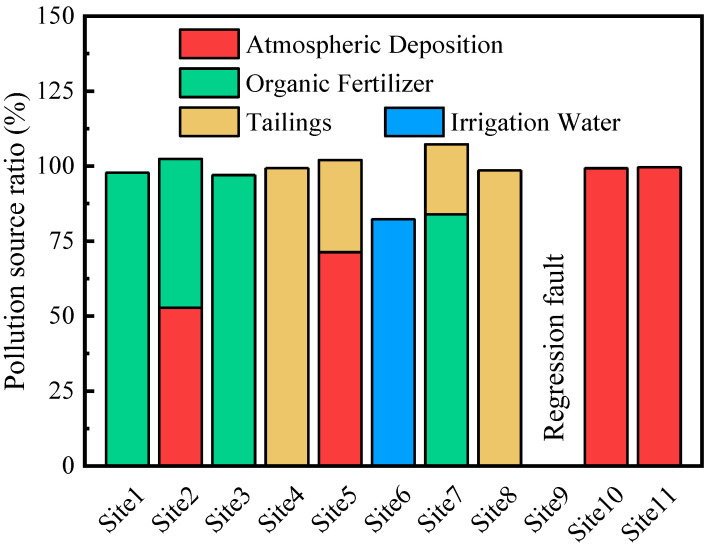
Contributions of different sources of pollution to different sites, determined using multiple linear regression.

The contribution of different pollution sources to each site was obtained using stepwise linear regression (Figure 6). For Site1 and Site3, the contributions of organic fertilizer were 97.8% (R^2^ = 0.948) and 97.0% (R^2^ = 0.929), respectively. For Site 10 and Site11, the contributions of atmospheric deposition were 99.3% (R^2^ = 0.983) and 99.6% (R^2^ = 0.991), respectively. For Site4 and Site8, the contributions of tailings were 99.4% (R^2^ = 0.987) and 98.6% (R^2^ = 0.966), respectively. For Site6, the contribution of irrigation water was 82.3% (R^2^ = 0.612). For Site2, the contributions of atmospheric deposition and organic fertilizer were 52.8% and 49.6% (R^2^ = 0.992), respectively. For Site5, the contributions of atmospheric deposition and tailings were 71.3% and 30.7% (R^2^ = 0.985), respectively. For Site7, the contributions of organic fertilizer and tailings were 83.9% and 23.4% (R^2^ = 0.992), respectively. Although the regression calculation for Site9 failed, the previous analysis suggests that the pollution source at this site may be associated with irrigation water.

The application of organic fertilizers is the secondary source of heavy metal pollution in China [4]. Organic fertilizer was applied at Site1, Site2, Site3, and Site7. Site3, in particular, is known as an area that is seriously contaminated with heavy metals and polluted with livestock and poultry feces [54]. The source identification method used in this study also indicated an extremely high contribution of organic fertilizers at this site, which is consistent with the large and frequent applications of organic manure. Although Site2 is located in an organic fertilizer polluted area, atmospheric deposition accounted for half of the contribution, which is similar to a previous report using an input inventory [7]. Road transportation is a mechanism that increases pollution by heavy metals near tailing sites [55]. Site4 was located close to a road and could easily be polluted by leakage from transported mining products. Site10 and Site11 were close to industrial or Pb-Zn mining areas and had high contributions of contamination from atmospheric deposition; this is consistent with previous findings from heavy metal input inventory calculations [7]. Site5 was not located close to any specific pollution sources; however, it showed extremely high heavy metal pollution from atmospheric deposition, providing evidence to support the great and widespread contribution of atmospheric deposition to the heavy metal pollution of soil in China [4]. Therefore, we can conclude that our method of source identification provided results consistent with the characteristics of pollution and rules of contaminant transport in the study area.

Until recently, the most common simple multivariate statistical methods, such as PCA, CA, and FA, required large samples and could not determine how individual sources contributed to pollution. For example, recent developments allowed 131 samples of surface soil (0–20 cm), collected from farmlands in 31 provinces, municipalities, and autonomous regions in the seven regions of China, to be analyzed via a combination of interelement relationships and PCA. The results showed that soil organic matter, measured in terms of the application of organic fertilizer, sewage sludge, and other organic materials, had a strong relationship with heavy metals such as Pb, Cd, Cr, Zn, Cu, and Sb, which are of an anthropogenic origin, whereas elements such as Mn, Ca, and Mg mainly originated from lithogenic sources [50]. GIS mapping requires abundant data on heavy metal concentrations, which can be difficult to obtain [23], and can only discriminate between the relative contributions of human input and natural background levels of an element of interest in soil. Lv, et al. [56] collected 646 samples in Ju Country in southeastern Shandong province, Eastern China. The results showed that Hg, Cd, Cu, Pb, and Zn were the main metals affected by human inputs therein, and a high risk of pollution resulting from an anthropogenic influence mainly occurred around urban areas in a pattern consistent with the spatial distribution of industrial sites and areas of heavy traffic. A previous study reported that a flag element ratio method does not require complex parameters and mathematical calculations and hence could relatively easily track sources of heavy metals on urban roads; however, a relatively large sample size is still needed [57].

Quantitative methods, such as PCA-related methods, PMF, and the isotope method, are also used to conduct pollutant source identification in soils. PCA-related methods need large amounts of data [12]. PMF has been widely applied to source apportionment of pollutants in the atmosphere [58], water [59], and sediments [60]. There are also some cases in which source apportionment was successfully used to determine the concentrations of heavy metals in soils via PMF [11,61], even though this failed to successfully apportion the contributions from three sources in the northwest corner of Zhejiang Province, China. This was attributed to the high skewness or outliers in the soil contamination data and the influence of the distance from each contributing source [62]. Isotope methods require complex preparations, such as digestion and purification procedures, and the accuracy of isotopic determination also affects the precision of the isotope ratios obtained [63]. Currently, Pb isotope analysis is the most commonly used isotope analysis method; however, the isotopic methods for soils, which are currently limited to Pb, Cd, Cu, Hg, and Zn, can only identify fewer than three pollution sources [18,19,20,21,23]. 

Our source identification method was based on the behavior of elements in the environment; it assumed that heavy metals with anthropogenic origins accumulate in the surface soils. Different ratios of accumulated elements were used in place of the different proportions of isotopes measured in the isotope source analysis method, which was a source of inspiration. The complex experimental operations used in the isotope source identification method were replaced by a convenient and straightforward analysis of heavy metal concentrations. This study shows that using the CA and PCA, along with MLR, can yield superior results for an analysis of more than 10 sites. Even if there are fewer sampling sites, and the CA and PCA cannot be performed, the pollution sources can still be analyzed via visual radar plots and MLR. The method proposed in this study overcomes the extensive data requirement and can easily analyze the sources of heavy metals in single or small plots.

## 4. Conclusions

The sources of heavy metal polluted soil at 11 sites in the Changsha–Zhuzhou–Xiangtan urban agglomeration were investigated using a relatively simple method for apportioning the pollution sources using a small sample size, simple experimental procedure, and convenient analysis technique. Four of the most common heavy metals (Cd, Cu, Pb, and Zn) in the region were used as research objects to identify pollution sources. The concentrations of all heavy metals were higher in surface soils compared to deep soils. Therefore, the concentrations of heavy metals accumulated in the surface soils were calculated as the difference between their concentrations in the surface and bottom soils. To identify the sources of pollution, radar plots were used for visually comparing and dividing sites of pollution into four different groups based on the source of pollution (atmospheric deposition, organic fertilizer, irrigation water, and tailings). An MLR model successfully calculated the contributions of different pollution sources, and the results were consistent with those expected for the test sites compared with known pollution sources. This method does not require an extensive dataset or complex experimental procedures; moreover, it is advantageous in that it can identify the sources of heavy metals in single or small plots. This study can be considerably significant in controlling sources of pollution and remediating small-scale soil pollution.

## Figures and Tables

**Table 1:** Basic information of sampling sites.

Sampling Site	Location	Possible Pollution Sources and Status
Site1	Changsha	organic fertilizer
Site2	Changsha	organic fertilizer
Site3	Changsha	livestock and poultry waste
Site4	Changsha	road dust
Site5	Changsha	ambiguous
Site6	Changsha	ambiguous
Site7	Zhuzhou	industrial pollution
Site8	Xiangtan	industrial pollution
Site9	Zhuzhou	lead-zinc mining
Site10	Zhuzhou	lead-zinc mining

**Table 2:** Concentrations of heavy metals from different pollution sources.

Pollution Source	Heavy Metals			
	Cd	Cu	Pb	Zn
Atmospheric Deposition (ng m^−3^)	5.28 ± 3.58	41.5 ± 4.0	121.5 ± 48.9	288.7 ± 214.0
Organic Fertilizer (mg kg^−1^)	0.59 ± 0.42	166.4 ± 116.1	27.2 ± 16.2	671.9 ± 297.0
Tailings (mg kg^−1^)	46.6 ± 42.0	423.5 ± 285.5	8706.7 ±1885.8	6195.7 ± 2427.7
Irrigation Water (μg L^−1^)	2.50 ± 41.6	4.18 ± 2.39	2.77 ± 3.67	39.2 ± 4.26

References for data sources: [7,28,31,32,33,34,35,36,37,38,39,40,41].

## Data Availability

Not applicable
